# GliPR1 knockdown by RNA interference exerts anti‐glioma effects in vitro and in vivo

**DOI:** 10.1007/s11060-021-03737-3

**Published:** 2021-04-15

**Authors:** Urban J. Scheuring, Steffi Ritter, Daniel Martin, Gabriele Schackert, Achim Temme, Stefanie Tietze

**Affiliations:** 1 Department of Hematology/Oncology and Infectious Diseases, University Hospital, J.W. Goethe University, Theodor-Stern-Kai 7, 60590 Frankfurt am Main, Germany; 2 Department of Neurosurgery, Section Experimental Neurosurgery/Tumor Immunology, University Hospital Carl Gustav Carus, Technical University Dresden, Fetscherstrasse 74, 01307 Dresden, Germany; 3 German Cancer Consortium (DKTK), Dresden, Germany; 4 German Cancer Research Center (DKFZ), Heidelberg, Germany; 5 National Center for Tumor Diseases, University Hospital Carl Gustav Carus, Technical University Dresden, Dresden, Germany

**Keywords:** GliPR1, Knockdown, RNA interference, Glioblastoma, Tumor growth, Survival

## Abstract

**Introduction:**

In human glioblastomas, glioma pathogenesis-related protein1 (GliPR1) is overexpressed and appears to be an oncoprotein. We investigated whether GliPR1 knockdown in glioma cells by RNA interference exerts anti-glioma effects.

**Methods:**

Experiments used human glioblastoma cell lines transduced with GliPR1 shRNA (sh#301, sh#258). Transduction produced stringent doxycycline-dependent GliPR1 knockdown in clones (via lentiviral “all-in-one” TetOn-shRNA vector) or stable GliPR1 knockdown in polyclonal cells (via constitutive retroviral-shRNA vector). In vitro assessments included cellular proliferation and clonogenic survival. In vivo assessments in tumor-bearing nude mice included tumor growth and survival.

**Results:**

Using doxycycline-dependent GliPR1 knockdown, shGliPR1-transduced U87-MG clones demonstrated reductions in cellular proliferation in the presence versus absence of doxycycline. Using stable GliPR1 knockdown, polyclonal shGliPR1-transduced U87-MG, A172, and U343-MG cells consistently showed decreased clonogenic survival and induced apoptosis (higher proportion of early apoptotic cells) compared to control shLuc-transduced cells. In tumor-bearing nude mice, using doxycycline-dependent GliPR1 knockdown, subcutaneous and cranial transplantation of the U87-MG clone 980-5 (transduced with GliPR1 sh#301) resulted in reduced subcutaneous tumor volume and cerebral tumor area in doxycycline-treated mice versus those left untreated. Using stable GliPR1 knockdown, nude mice cranially transplanted with polyclonal U87-MG cells transduced with GliPR1 sh#258 had significantly prolonged survival compared to mice cranially transplanted with control shLuc-transduced cells (41 versus 26 days; *P* < 0.001).

**Conclusion:**

GliPR1 knockdown in glioma cells decreased cellular proliferation, decreased clonogenic survival, and induced apoptosis in vitro, and reduced glioblastoma tumor growth and prolonged survival in vivo. These findings support that GliPR1 may have potential value as a therapeutic target.

**Supplementary Information:**

The online version contains supplementary material available at 10.1007/s11060-021-03737-3.

## Introduction

Glioblastoma is the most frequent and aggressive primary brain tumor in adults and the prognosis remains poor [1]. The therapeutic standard comprises surgical resection, followed by radio-chemotherapy plus temozolomide and temozolomide maintenance [2, 3]. Although promising modern compounds have entered clinical trials [4, 5], none has yet led to regulatory approval of a new therapy.

Glioma pathogenesis-related protein1 (GliPR1) was originally identified in human glioblastoma [6] and is also named *related to testes-specific, vespid, and pathogenesis protein 1* (RTVP-1) [7]. Whilst in glioblastoma and some other tumors (including Wilm’s tumor, acute myeloid leukemia, invasive melanoma) GliPR1 is overexpressed and appears to be an oncoprotein that enhances proliferation [8–11], in other tumors (including prostate, lung and bladder cancers, sarcoma, multiple myeloma) it appears to be a tumor suppressor and to induce apoptosis [12–21]. GliPR1 has also been identified as a marker of myelomonocytic differentiation in macrophages [22] and as an HIV-1 dependency factor [23].

At the same time that mechanisms underlying GliPR1 regulation of glioma cell migration and invasion were being elucidated [24, 25], we were interested to assess its value as a therapeutic target in glioblastoma. We conducted in vitro and in vivo experiments to investigate whether RNA interference targeting GliPR1, via transduction of small-hairpin RNA (shRNA) sequences, causes knockdown of its gene and protein expression and exerts anti-glioma effects.

## Results

### Doxycycline-dependent knockdown of GliPR1 in vitro in glioma cell clones

**Fig. 1:**
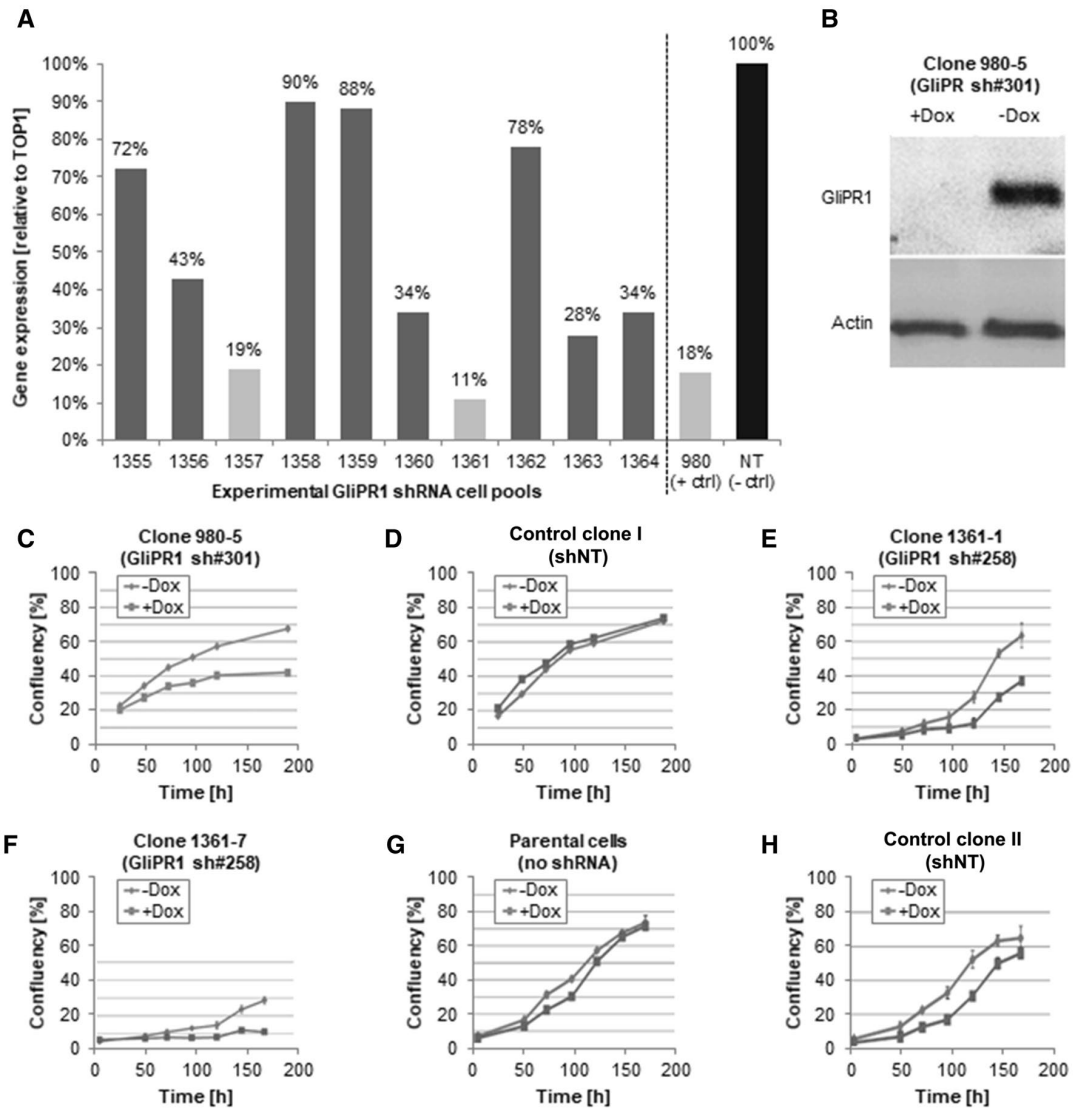
Doxycycline-dependent GliPR1 knockdown in U87-MG glioma cells. **a** GliPR1 gene expression (measured by qRT-PCR) in U87-MG cell pools transduced with various GliPR1 shRNAs or a non-targeting control shRNA (shNT) using a lentiviral “all-in-one” TetOn-shRNA vector. In this preliminary experiment, GliPR1 shRNAs were compared to find the most potent knockdown sequences, in the presence of doxycycline. The GliPR1 sh#301 (in cell pool 980) had already been established as potent in an earlier experiment. The GliPR1 sh#258 (in cell pool 1361) was found to be the most potent. Data are mean relative to TOP1 reference gene expression, in the absence of doxycycline. **b** GliPR1 protein expression (measured by Western blot) of clone 980-5 (transduced with GliPR1 sh#301), in presence and absence of doxycycline. Actin is loading control. **c–d** Confluency assays of clone 980-5 (transduced with GliPR1 sh#301) and a control clone (transduced with shNT), in presence and absence of doxycycline (using CloneSelect Imager), over time. Data are mean. **e–h** Confluency assays of clones 1361-1 and 1368-7 (transduced with GliPR1 sh#258), polyclonal parental U87-MG cells, and control clones (transduced with shNT), in presence and absence of doxycycline (using CloneSelect Imager), over time. Data are mean ± SD

We first transduced the U87-MG human glioblastoma cell line with GliPR1 shRNAs (shGliPR1s) using a lentiviral “all-in-one” TetOn-shRNA vector, which produces stringent doxycycline-dependent knockdown. In preliminary experiments, we generated U87-MG cell pools transduced with various shGliPR1 sequences to find the most potent knockdown sequences. A non-targeting shRNA (shNT) served as a negative control. As shown in Fig. 1a, transductions of GliPR1 sh#301 (in cell pool 980) and GliPR1 sh#258 (in cell pool 1361) led to the greatest reductions in GliPR1 gene expression in the presence of doxycycline (relative to TOP1 reference gene expression in the absence of doxycycline), to 18 % and 11 %, respectively.

We went on to establish clones from these U87-MG cell pools and found that, in the presence of doxycycline, clones 980-5 (transduced with GliPR1 sh#301) and 1361-1 and − 7 (transduced with GliPR1 sh#258) exhibited high levels of GliPR1 gene expression knockdown (data not shown) and efficient GliPR1 protein expression knockdown (exemplar shown in Fig. 1b), each at 72 h after doxycycline treatment start. In confluency assays conducted over 7 days, these shGliPR1-transduced clones demonstrated reductions in cellular proliferation in the presence versus absence of doxycycline at all time points tested (Fig. 1c–h). Polyclonal parental U87-MG cells and shNT-transduced control clones did not show a doxycycline-induced reduction in cellular proliferation.

### Stable knockdown of GliPR1 in vitro in polyclonal glioma cells

To limit any positional effects of provirus, we then established polyclonal glioma cells with stable GliPR1 knockdown, by transducing three human glioblastoma cell lines (U87-MG, A172, U343-MG) with each of GliPR1 sh#301 and GliPR1 sh#258 using a constitutive retroviral-shRNA vector. A previously described luciferase shRNA (shLuc) [26] served as a negative control.

**Fig. 2:**
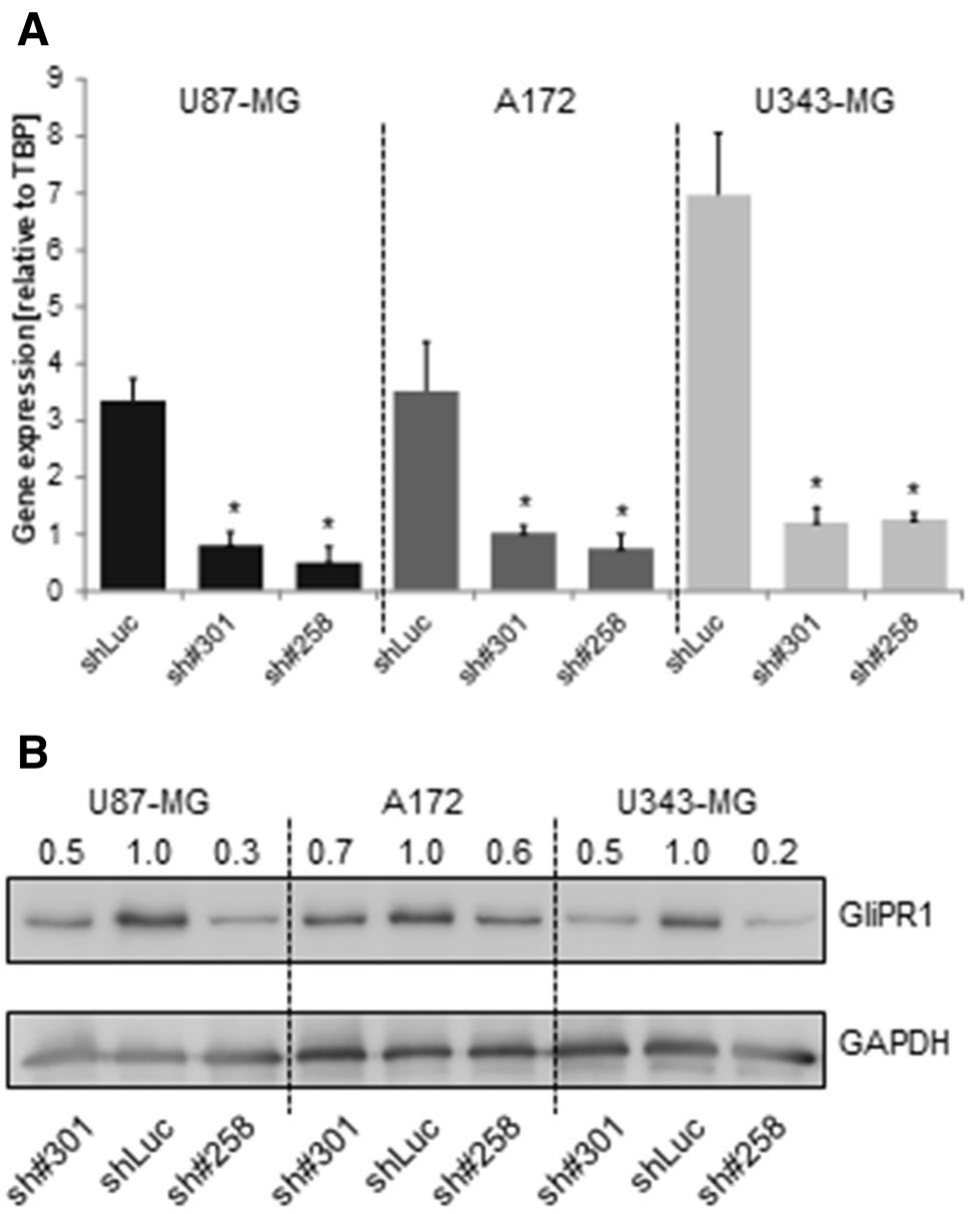
Stable GliPR1 knockdown in polyclonal U87-MG, A172, and U343-MG glioma cells transduced with GliPR1sh#301, GliPR1sh#258, or a control luciferase shRNA (shLuc), at 7 days post transduction. **a** GliPR1 gene expression by qRT-PCR. Data are mean ± SD relative to TBP reference gene expression. *P < 0.05 versus shLuc control in same cell line. **b** GliPR1 protein expression by Western blot. Data are mean relative to GliPR1 protein expression for shLuc control in same cell line. GADPH is loading control

**Fig. 3:**
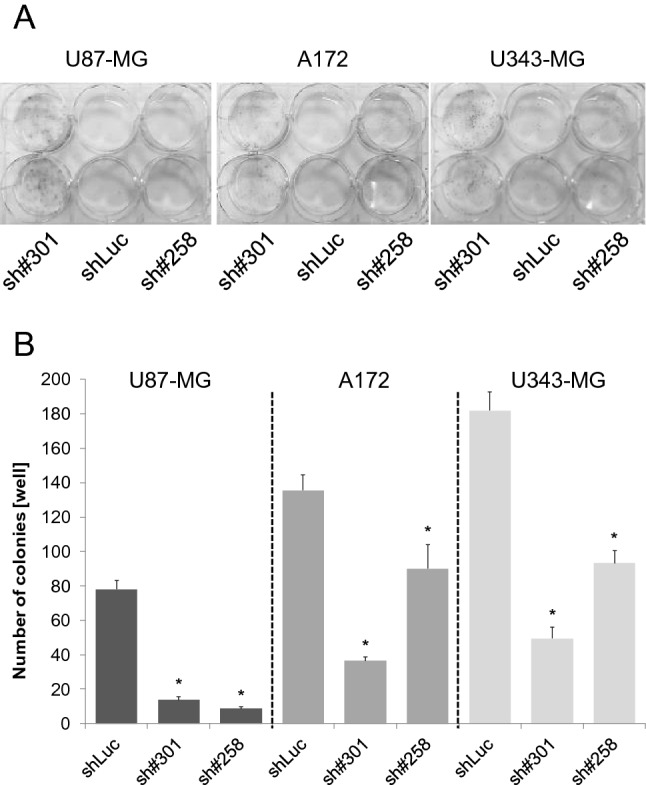
Clonogenic survival following stable polyclonal GliPR1 knockdown in U87-MG, A172, and U343-MG glioma cells transduced with GliPR1sh#301, GliPR1sh#258, or a control luciferase shRNA (shLuc), at 3 weeks post transduction. **a** Representative images showing clonogenic survival results. **b** Quantification of clonogenic survival results. Data are mean ± SD number of colonies per well. *P < 0.05 versus shLuc control in same cell line

**Fig. 4:**
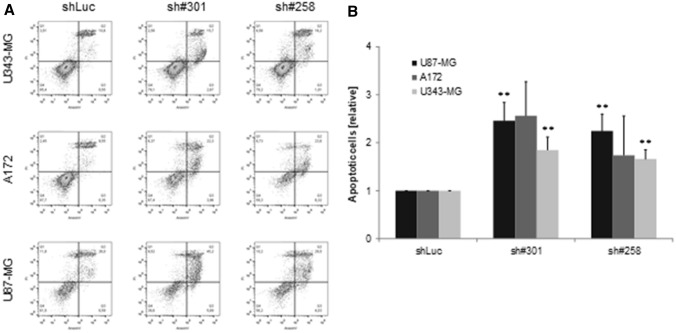
Induction of apoptosis following stable polyclonal GliPR1 knockdown in U87-MG, A172, and U343-MG glioma cells transduced with GliPR1sh#301, GliPR1sh#258, or a control luciferase shRNA (shLuc), at 7 days post transduction. **a** Flow cytometry dot plot of cells stained using Annexin V FITC/PI kit, which distinguishes early apoptotic cells (annexin V positive, PI negative) from late apoptotic/necrotic cells (PI positive) and viable cells (annexin V negative, PI negative). **b** Quantification of apoptosis results. Data are mean ± SD proportion of apoptotic cells relative to shLuc control in same cell line. **P < 0.001 for U87-MG, and U343-MG glioma cells transduced with GliPR1sh#301, GliPR1sh#258, or a control luciferase shRNA (shLuc)

At 7 days post transduction, in all three cell lines, GliPR1 gene expression levels (relative to TBP reference gene expression) were significantly decreased for each shGliPR1 transduction when compared to shLuc control transduction (*P* < 0.05 in each case) (Fig. 2a). Efficient GliPR1 protein expression knockdown was also achieved in all cell lines for each shGliPR1 transduction (Fig. 2b): GliPR1 protein levels were significantly decreased versus shLuc control transduction by 30–50 % in U87-MG cells, 60–70% in A172 cells, and 20–50% in U343-MG cells (*P* < 0.05 in each case). In clonogenic survival assays, in all cell lines, the number of colonies per well was significantly fewer at 3 weeks post transduction for each shGliPR1 transduction versus shLuc control transduction (*P* < 0.05 in each case) (Fig. 3). An apoptosis assay, where cells in early apoptosis displayed annexin V-positive characteristics by flow cytometry, showed that at 7 days post transduction, in all cell lines, the proportion of early apoptotic cells was higher for each shGliPR1 transduction versus shLuc control transduction (Fig. 4).

### In vivo effects of doxycycline-dependent GliPR1 knockdown on glioblastoma tumor growth

First experiments in nude mice (NMRI:nu/nu) explored effects of stringent doxycycline-dependent GliPR1 knockdown on glioblastoma tumor growth, using U87-MG clone 980-5 transduced with GliPR1 sh#301.

**Fig. 5:**
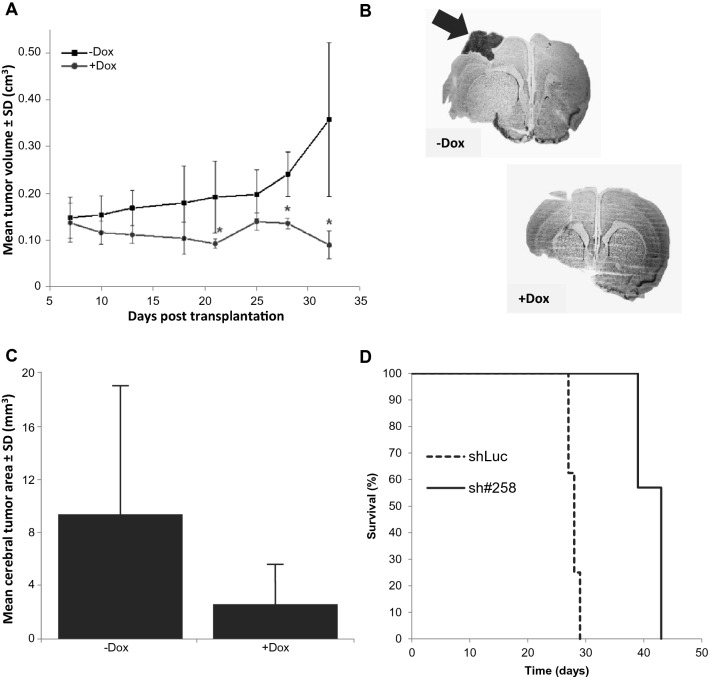
In vivo effects of GliPR1 knockdown in U87-MG glioma cells. **a, b** Tumor growth upon doxycycline-dependent GliPR1 knockdown, using clone 980-5 transduced with GliPR1 sh#301. 8 mice underwent subcutaneous transplantation and 4 additionally underwent cranial transplantation; from day 8, 4 were left untreated (-Dox) and 4 were given doxycycline in drinking water (+ Dox). **a** Subcutaneous tumor volumes over time. Data are mean ± SD. *P < 0.05 for + Dox group versus -Dox group. **b** Representative cryosections (stained with cresyl violet) of brains from mice in -Dox group and + Dox group. Arrow shows cerebral tumor at end of experiment in -Dox example. **c** Cerebral tumor area at end of second experiment with doxycycline-dependent GliPR1 knockdown (clone 980-5; sh#301) using larger cohorts (each n = 5). Data are mean ± SD. *P* = 0.056 for + Dox group versus -Dox group. **d** Survival upon stable GliPR1 knockdown, using polyclonal U87-MG cells transduced with GliPR1 sh#258 (n = 14) or a control luciferase shRNA (shLuc; n = 16). Kaplan-Meier plot. *P* < 0.001 for between-group difference

In an initial experiment, 8 mice underwent subcutaneous (left flank) transplantation of clone 980-5 and 4 of these additionally underwent cranial (right brain hemisphere) transplantation. From day 8 onwards, 4 mice were given doxycycline in their drinking water to induce GliPR1 knockdown and 4 mice were left untreated. Serial measurements of the subcutaneous tumor volumes at days 7, 10, 13, 18, 21, 25, 28, and 32 post transplantation revealed suppression in the doxycycline-treated group compared to the untreated group, that was statistically significant (*P* < 0.05) at days 21, 28, and 32 (Fig. 5a). The difference was greatest at day 32, when the subcutaneous tumor volume was mean 0.089 ± 0.031 cm^3^ in the doxycycline-treated group versus 0.363 ± 0.168 cm^3^ in the untreated group (each n = 4), representing a ratio of 26.5 %. That clone 980-5 was capable of establishing intracranial tumors was confirmed by histological analyses of brain sections (Fig. 5b). Furthermore, doxycycline treatment apparently induced cerebral tumor regression.

A further experiment using larger cohorts (each n = 5) was performed to validate therapeutic effects mediated by knockdown of GliPR1. Among mice transplanted with clone 980-5 and subsequently treated with doxycycline, the mean cerebral tumor area measured from histological brain sections was 2.59 ± 3.00 mm^2^, which was lower (*P =* 0.056) than the mean cerebral tumor area of 9.34 ± 9.49 mm^2^ in the untreated group (Fig. 5c). This experiment confirmed the anti-tumor effect of doxycycline-induced GliPR1 knockdown. Unexpectedly, when analyzing tumors derived from shNT-transduced U87-MG clone we observed that doxycycline treatment itself exerted some inhibitory effect on tumor growth (data not shown).

### In vivo effects of stable GliPR1 knockdown on survival in tumor-bearing nude mice

To avoid any potential anti-glioma effect of doxycycline, and to minimize positional effects of shRNA-expressing provirus, in vivo effects of stable GliPR1 knockdown in tumor-bearing nude mice were further investigated using polyclonal U87-MG cells transduced with the GliPR1 sh#258. Polyclonal U87-MG cells transduced with shLuc served as a negative control. In a survival experiment, mice underwent cranial (right brain hemisphere) transplantation with GliPR1 sh#258-transduced cells (n = 14) or control shLuc-transduced cells (n = 16). That polyclonal U87-MG cells used were capable of establishing intracranial tumors was confirmed by histological analyses of brain sections (Figure S1). Median survival was 41 versus 26 days (*P* < 0.001) (Fig. 5d). This experiment thus confirmed that the anti-tumor effect seen with doxycycline-induced GliPR1 knockdown also occurs with stable GliPR1 knockdown (i.e. in the absence of any doxycycline effect).

## Discussion

Our in vitro findings in glioma cells provide evidence of anti-glioma effects of GliPR1 knockdown by RNA interference. First, transduction of a human glioblastoma cell line with each of two shGliPR1s (sh#301 and sh#258) via a lentiviral “all-in-one” TetOn vector enabled establishment of glioma cell clones exhibiting stringent doxycycline-dependent knockdown of GliPR1 gene and protein expression. These clones demonstrated reductions in cellular proliferation in the presence versus absence of doxycycline. Second, transduction of three human glioblastoma cell lines with the same two shGliPR1s via a constitutive retroviral-shRNA vector enabled establishment of polyclonal glioma cells exhibiting stable knockdown of GliPR1 gene and protein expression. The shGliPR1-transduced cells consistently showed decreased clonogenic survival and induced apoptosis (with a higher proportion of early apoptotic cells) compared to the same cell line transduced with a control shRNA (shLuc). These in vitro findings are in line with those from another research group, who reported decreased cellular proliferation and induced apoptosis in glioma cells following GliPR1 knockdown mediated by transfection of a small interfering (si)RNA against GliPR1 [11]. The same group elucidated that GliPR1 appears to regulate glioma cell migration and invasion via interacting with the actin polymerization regulator N-WASP and its association with the ribonucleoprotein hnRNPK [11, 25], and to promote mesenchymal transformation via a STAT3/IL-6-dependent positive feedback loop [24]. It is unclear why GliPR1 manifests the apparent paradox that in glioblastoma and some tumors it is overexpressed and appears to be an oncoprotein [8–11] yet in other tumors it has downregulated expression and appears to be a tumor suppressor [12–19, 21]. It has been postulated that epigenetic modulation by promoter methylation could be involved [10, 16, 17]. Bier et al. studied the methylation status of miR-137, a putative suppressor miRNA, in GBM and GSCs. They found that in addition to significantly reduced miR-137 expression in GBM and GSCs compared to normal brains and neural stem cells (NSCs), the miR-137 promoter was hypermethylated in the GBM samples [27]. Interestingly, a study in metastatic melanoma cell lines found an association between decreasing promoter methylation and increasing GliPR1 gene expression, and siRNA-mediated GliPR1 knockdown in melanoma cells decreased cellular proliferation [8], similar to glioma cells.

Our in vivo findings provide evidence that GliPR1 knockdown by RNA interference in glioma cells translates into reduced glioblastoma tumor growth and prolonged survival in tumor-bearing nude mice. In two experiments using our doxycycline-dependent GliPR1 knockdown system, whereby mice underwent subcutaneous and cranial transplantation of a shGliPR1-transduced clone (clone 980-5 transduced with GliPR1 sh#301), those mice treated with doxycycline to induce GliPR1 knockdown had reduced subcutaneous tumor volume and cerebral tumor area compared to those left untreated. Of note though, our second experiment included control groups transplanted with an shNT-transduced clone or polyclonal parental cells, and results with these controls revealed that doxycycline treatment itself may have an inhibitory effect to glioblastoma tumor growth, at least in this mouse model in conjunction with these conditions. This is in line with subsequent reports of anti-glioma effects of doxycycline in glioma cell experiments [28, 29]. To avoid any potential anti-glioma effect of doxycycline and to furthermore limit positional effects of provirus compared to the use of cell clones, we decided to change our in vivo strategy and to employ our polyclonal, stable GliPR1 knockdown system (without requirement for doxycycline induction) for use in a survival study. The mice cranially transplanted with glioma cells transduced with an shGliPR1 (sh#258) achieved significantly prolonged survival compared to those cranially transplanted with glioma cells transduced with the control shLuc (*P* < 0.001), although a minor fraction of polyclonally transduced U87-MG cells had weaker RNAi effects due to a lower number or positioning of proviruses. This evidence of prolonged survival associated with GliPR1 knockdown in glioma cells is supported by another study in nude mice using xenografts derived from glioma stem cells (GSCs): mice cranially transplanted with xenografts of GSCs transduced with an shGliPR1 had significantly prolonged survival compared to a control group cranially transplanted with xenografts of GSCs transduced with a control shRNA (*P* < 0.001) [24].

In conclusion, we found that GliPR1 knockdown in glioma cells by RNA interference decreased cellular proliferation, decreased clonogenic survival, and induced apoptosis in vitro, and reduced glioblastoma tumor growth and prolonged survival in vivo in tumor-bearing nude mice. No clinical study so far has evaluated inhibition of GliPR1 overexpression as a therapeutic strategy in glioblastomas. Taken together with other laboratory findings [11, 24, 25], our observed anti-glioma effects of GliPR1 knockdown support that GliPR1 may have potential value as a therapeutic target for this aggressive brain tumor with a poor prognosis.

## Materials and methods

### Cell culture

U87-MG, A172, and U343-MG human glioblastoma-derived cell lines (from ATCC or H.K. Schackert, University Hospital Carl Gustav Carus, Dresden, Germany) and human embryonic kidney 293T cells (from ATCC or D. Lindemann, Technical University Dresden, Germany) were authenticated (Multiplex Cell Line Authentication test, Multiplexion, Friedrichshafen, Germany).

Investigations of doxycycline-dependent GliPR1 knockdown used U87-MG clones transduced with an shGliPR1, or shNT control, via a lentiviral “all-in-one” TetOn vector. Cell culture used MEM. For quantitative reverse transcriptase polymerase chain reaction (qRT-PCR) and Western blots, clones were seeded at 10^5^ cells/well into 6-well plates, the next day either left untreated or treated with 1 µg/ml doxycycline (Merck KgaA, Darmstadt, Germany), and harvested at 72 h after doxycycline start. For confluency assays, clones were seeded at 10^4^ cells/well into 12-well plates and the next day either left untreated or treated with 1 µg/ml doxycycline; confluency was assessed over 7 days using the CloneSelect Imager (Molecular Devices, LLC, San José, CA, USA).

Investigations of stable GliPR1 knockdown used the three glioma cell lines transduced polyclonally with an shGliPR1, or previously described shLuc control [26], via a constitutive retroviral-shRNA vector. Cell culture used DMEM containing 4.5 mg/l glucose, 10 % FCS, penicillin/streptomycin, and 10 mM Hepes. For qRT-PCR, Western blots, and apoptosis analyses, cells were seeded at 10^5^ cells/well into 6-well plates, with transduction 24 h later, and harvesting at 7 days post transduction. For clonogenic survival assays, cells were seeded at 100 cells/well into 6-well plates, with transduction 24 h later, and after 3 weeks, clones were stained (0.2 % crystal violet) and counted macroscopically.

Experiments used at least triplicates and were performed at least twice.

### Synthesis of shRNAs

shRNA synthesis used the self-inactivating retroviral Moloney murine leukemia virus backbone pRVH-1-puroR [30]. DNA oligonucleotides sh#301 and sh#258 against GliPR1 (Eurofins MWG Biotech, Ebersberg, Germany) were: #301 upper strand: 5′-*gatct*ccAGCCAGTGATATGCTATACATTTCAAGAGAATGTATAGCATATCACTGGCTTTTTTggag*c*-3′, #301 bottom strand: 5′-*gctc*cAAAAAGCGTTCGAATCCATAACAAGTTCTCTTGAAACTTGTTATGGATTCGAACGCgg*a*-3′; #258 upper strand: 5′-*gatct*ccGCGTTCGAATCCATAACAAGTTTCAAGAGAACTTGTTATGGATTCGAACGCTTTTTggag*c*-3′, #258 bottom strand: 5′-*gctcc*AAAAAGCGTTCGAATCCATAACAAGTTCTCTTGAAACTTGTTATGGATTCGAACGCgg*a*-3′. shNT was a negative control: upper strand: 5′-TGCTGTTGGTGCTCTTCATCTTGTTGGTTTTGGCCACTGACTGACCAACAAGAAAGAGCACCAA-3′, bottom strand: 5′- TTGGTGCTCTTTCTTGTTGGTCAGTCAGTGGCCAAAACCAACAAGATGAAGAGCACCAACAGCA-3′. shLuc [26] was also a negative control. After annealing of the single strands, the shRNA-encoding fragment was ligated into the BglII/SalI-restrictions sites of pRVH-N1-puro.

### Preparation of shRNA vectors and transduction of glioma cells

Lentiviral (HIV-based, VSVG pseudotyped, self-inactivating) “all-in-one” TetOn-shRNA vectors (SIRION Biotech, Martinsried, Germany) were cotransfected into 293T cells with the plasmids pMDL, pRev, and pVSVG. Viral genomic titers were determined using the Lenti-X qRT-PCR titration kit (Clontech, Heidelberg, Germany). U87-MG cells were transduced with a multiplicity of infection of 5. Starting 48 h post transduction, cells were cultured for 2 weeks with 0.5 µg/mL puromycin (Gibco, Schwerte, Germany), and resulting transduced cell pools were frozen at 5 × 10^6^ cells/vial. For generation of clones, cell pools were seeded by limited dilution into 96-well plates; after 2 weeks, wells with a single clone were identified. These clones were expanded to 5 × 10^5^ cells, with half used for GliPR1 gene knockdown verification by qRT-PCR and the remainder frozen for experiments.

Constitutive retroviral-shRNA vectors [31] were cotransfected into 293T cells with an expression construct for gag-pol (pHIT60) and the vesicular stomatitis virus G-protein (pMD.G2). Virus-containing supernatants were harvested 48 h after transfection. For transductions, the virus-containing supernatants were added at 24 h after seeding, and transduced cells were selected using 10 µg/ml puromycin (Life Technologies, Darmstadt, Germany) for 24 h.

### Real‐time qRT-PCR analysis

In doxycycline-dependent GliPR1 knockdown experiments, total mRNA was prepared using the NucleoSpin RNA II kit (Macherey-Nagel, Düren, Germany). cDNA was generated using 1 µg total mRNA, oligo(dT)_12−15_ primer (GE Healthcare, Freiburg, Germany), and the Omniscript RT kit (Qiagen, Hilden, Germany). qRT-PCR was performed using the LightCycler 480 system (Roche, Basel, Switzerland). Primers were GliPR1-F 5′-ATGCGTGTCACACTTGCTACA-3′ and GliPR1-R 5′-TCACCTCTGATCGGAACTTGT-3′. Reference primers were TOP1-F 5′-CCAGACGGAAGCTCGGAAAC-3′ and TOP1-R 5′-GTCCAGGAGGCTCTATCTTGAA-3′.

In stable GliPR1 knockdown experiments, total mRNA was prepared using QiaShredder and the RNAeasy kit (Qiagen, Hilden, Germany). cDNA was generated using 1 µg total mRNA, oligo(dT)_12−15_ primer, and the Omniscript RT kit. qRT-PCR was by the PikoReal real-time PCR system (Thermo Fisher Scientific, Schwerte, Germany). Primers were GliPR1-F 5′-AGAGGTGAAACCAACAGCCAGT-3′ and GliPR1-R 5′-CAGCTTGTGGGGTGGCTTCA-3′. Reference primers [32] were TBP-F 5′-TGCACAGGAGCCAAGAGTGAA-3′ and TBP-R 5′-CACATCACAGCTCCCCACCA-3′.

Experiments used SYBR-Green. Amplification protocol was: 95 °C denaturation 10 min; then, 40 cycles at 95 °C denaturation 15 s, 61 °C annealing 5 s, and 72 °C extension 5 s.

### Western blots

In doxycycline-dependent GliPR1 knockdown experiments, Western blotting used Lämmli lysis buffer, 20 µg protein/lane, and 2 % ECL Advance blocking agent (GE Healthcare, Freiburg, Germany). Probes were a monoclonal mouse anti-GliPR1 (1:2000; #H00011010-M04; Abnova, Taipei, Taiwan) then horseradish peroxidase-conjugated anti-mouse antibody (Cell Signaling, Massachusetts, USA). Signals were detected with the ECL Plus detection system (GE Healthcare, Freiburg, Germany) and VersaDoc imager (Bio-Rad, Feldkirchen, Germany). Polyclonal anti-pan-actin antibody (Cell Signaling, Massachusetts, USA) verified equal loading.

In stable GliPR1 knockdown experiments, Western blotting used a lysis buffer (10 mM Tris-HCl pH 8.0, 140 mM NaCl, 1 % Triton-X-100), 30 µg protein/lane, and 5 % milk powder (Carl Roth GmbH & Co, Karlsruhe, Germany) or BSA (Sigma-Aldrich Chemie GmbH, Hamburg, Germany). Probes were the same monoclonal mouse anti-GliPR1 (1:2000) then horseradish peroxidase-conjugated anti-mouse antibody (Dako, Glostrup, Denmark). Signals were detected with the ECL Plus detection system and LAS-3000 imager (Fujifilm Life Sciences Strategy, Cambridge, USA). Monoclonal anti-actin or anti-GAPDH antibody (both Cell Signaling, Massachusetts, USA) verified equal loading.

### Apoptosis analysis

Apoptosis analysis, using cells from the culture supernatant and adherent cells (gently detached using collagenase IV), was by flow cytometry using the Annexin V-fluorescein isothiocyanate/propridium iodide kit (Miltenyi Biotec, Bergisch Gladbach, Germany). For calculations of induced cell death, percentage of annexin V-positive PBS-treated cell fraction was subtracted from percentage of shRNA-transduced cells. Signals were measured using the MACS-Quant flow cytometer (Miltenyi Biotec, Bergisch Gladbach, Germany) and analyzed with FlowJo software version 7.6.5 (Tree Star, Ashland, USA).

### Tumor growth and survival in nude mice

NMRI-(nu/nu)-nude mice aged 9–12 weeks (from Taconics, Bomholtgard, Germany or Medical Faculty, Technical University Dresden, Germany) were held under standardized pathogen-free conditions with *ad libitum* access to food and water. Numbers of mice per group are reported in the Results.

In first experiments, effects of stringent doxycycline-dependent GliPR1 knockdown on tumor growth used the U87-MG 980-5 clone transduced with GliPR1 sh#301, with experiments carried out by EPO GmbH (Berlin, Germany) using a published procedure [33] with minor modifications. Subcutaneous and cranial transplantations were undertaken in parallel: 1 × 10^7^ cells (in 100 µl PBS) subcutaneously transplanted into the left flank and 5 × 10^4^ cells (in 2 µl PBS) stereotactically transplanted into the right brain hemisphere. From day 8, drinking water contained 5 % sucrose with or without 6 mg/ml doxycycline (Genaxxon Bioscience, Ulm, Germany). Subcutaneous tumor volume was measured twice weekly in two dimensions with a caliber-like instrument and calculated as V = (length + [width]^2^)/2. All mice were sacrificed when respective control group became moribund. Brains were shock-frozen in 2-methylbutane, then sequential cryosections (10 μm) were stained with cresyl violet. Cerebral tumor area was determined in brain sections with the largest tumor extension using an Axioscope (Zeiss, Jena, Germany).

In a subsequent experiment, effects of stable GliPR1 knockdown on survival were investigated using polyclonal mCherry-tagged U87-MG cells transduced with GliPR1 sh#258 or control shLuc. Mice were randomized, balancing by weight and age. For cranial transplantation, 1 × 10^6^ cells (in 10 µl PBS) were stereotactically transplanted into the right brain hemisphere. Kaplan-Meier survival curves were constructed, and median survival calculated using PRISM software (GraphPad, San Diego, USA). Brains were shock-frozen in 2-methylbutane, then sequential cryosections (10 μm) were stained with Haematoxylin and Eosin (H&E), Hoechst 33,342 (Life Technologies, Carlsbad, USA), and for the proliferation marker Ki-67 (Agilent Technologies, Santa Clara, USA).

### Statistical analyses

In vitro experiments used Student’s *t* test for statistical analysis. In vivo experiments used Mann–Whitney U test for tumor growth and log-rank test for survival.

## Supplementary Information

Below is the link to the electronic supplementary material.

Fig. S1 Histology and immunohistochemistry of representative brain cryosections from tumor bearing mice after implantation of polyclonal mCherry-tagged U87-MG cells transduced with GliPR1 sh#258 or a control luciferase shRNA (shLuc). (a-b) Imaging for mCherry red fluorescent protein as surrogate marker for tumors (red) and counterstaining with Hoechst 33342 nucleic acid stain (blue). (c-d) Immunohistochemical staining for proliferation marker Ki-67 (brown). (e-f) Hematoxylin and eosin (H&E) staining shows high nuclear density in the tumor. Scale bar = 100 μm. Supplementary material 1 (PPTX 5689.6 kb)

## Data Availability

Primary experimental data and materials are available upon request from the corresponding authors: stefanie.tietze@uniklinikum-dresden.de and u.scheuring@gmx.de.
