# Supplementary figures and images for: GliPR1 knockdown by RNA interference exerts anti‐glioma effects in vitro and in vivo
Source: J Neurooncol. 2021 Apr 15;153(1):23–32. doi: 10.1007/s11060-021-03737-3 (PMC8131343; doi:10.1007/s11060-021-03737-3)

## Slide 1
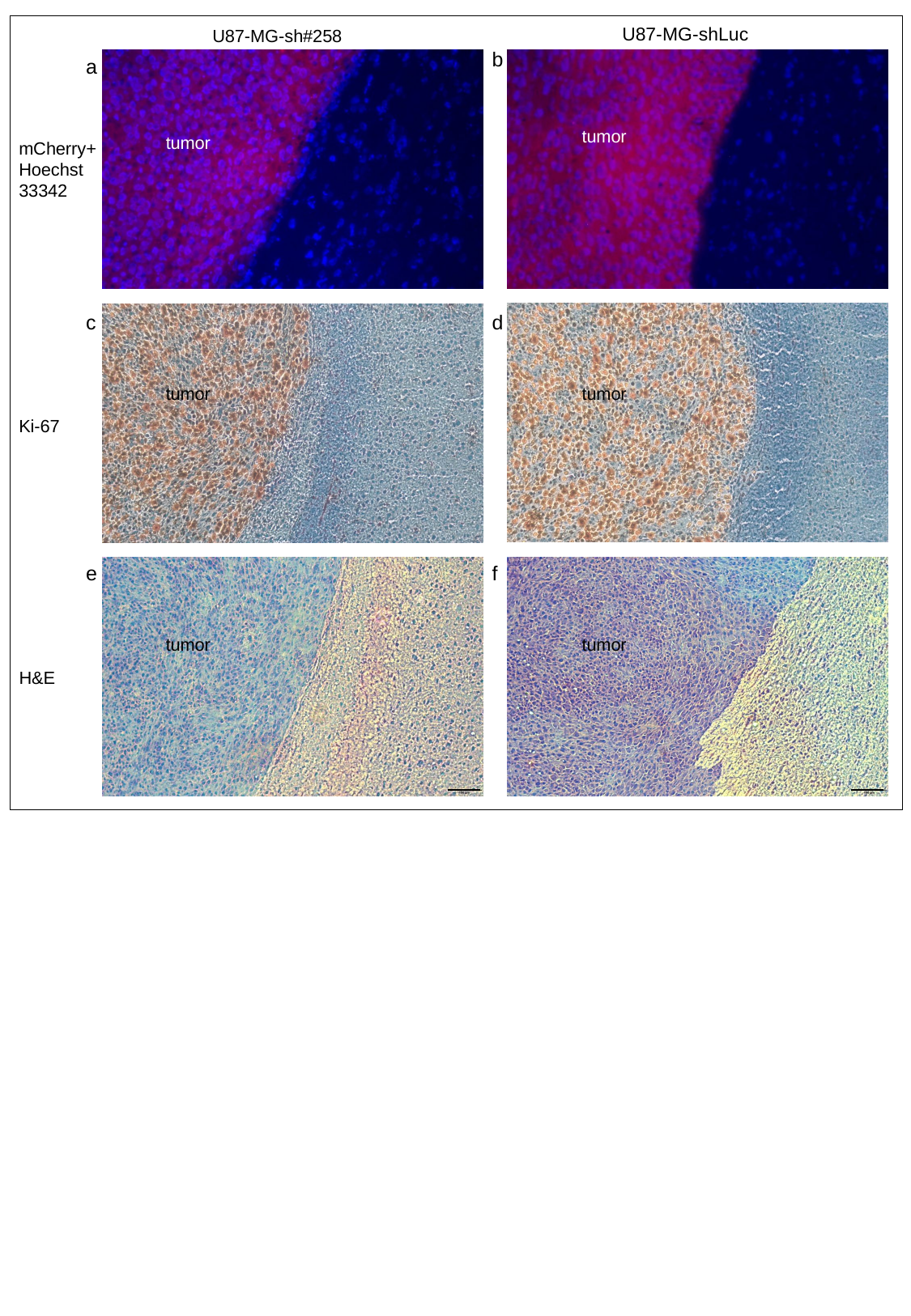

U87-MG-shLuc
U87-MG-sh#258
b
a
tumor
tumor
mCherry+ Hoechst 33342
c
d
tumor
tumor
Ki-67
e
f
tumor
tumor
H&E

Supplement: Supplementary file 1 — Histology and immunohistochemistry of representative brain cryosections from tumor bearing mice after implantation of polyclonal mCherry-tagged U87-MG cells transduced with GliPR1 sh#258 or a control luciferase shRNA (shLuc). (a-b) Imaging for mCherry red fluorescent protein as surrogate marker for tumors (red) and counterstaining with Hoechst 33342 nucleic acid stain (blue). (c-d) Immunohistochemical staining for proliferation marker Ki-67 (brown). (e-f) Hematoxylin and eosin (H&E) staining shows high nuclear density in the tumor. Scale bar = 100 μm. Supplementary material 1 (PPTX 5689.6 kb) [file 11060_2021_3737_MOESM1_ESM.pptx]
